# Advances in the treatment of Adamantinomatous craniopharyngioma: How to balance tumor control and quality of life in the current environment: a narrative review

**DOI:** 10.3389/fonc.2023.1326595

**Published:** 2023-12-21

**Authors:** Ao Chen, MingDa Ai, Tao Sun

**Affiliations:** ^1^ Department of Neurosurgery, Yueyang People’s Hospital, Yueyang, China; ^2^ Department of Neurosurgery, The First Affiliated Hospital of Kunming Medical University, Kunming, China

**Keywords:** Adamantinomatous craniopharyngioma (ACP), cystic craniopharyngioma, quality of life, cyst management, tumor control

## Abstract

Adamantinomatous craniopharyngioma (ACP) presents a significant challenge to neurosurgeons despite its benign histology due to its aggressive behavior and unique growth patterns. This narrative review explores the evolving landscape of ACP treatments and their efficacy, highlighting the continuous development in therapeutic approaches in recent years. Traditionally, complete resection was the primary treatment for ACP, but surgical -related morbidity have led to a shift. The invasive nature of the finger-like protrusions in the histological structure results in a higher recurrence rate for ACP compared to papillary craniopharyngioma (PCP), even after complete macroscopic resection. Given this, combining subtotal resection with adjuvant radiotherapy has shown potential for achieving similar tumor control rates and potentially positive endocrine effects. Simultaneously, adjuvant treatments (such as radiotherapy, intracystic treatment, and catheter implantation) following limited surgery offer alternative approaches for sustained disease control while minimizing morbidity and alleviating clinical symptoms. Additionally, advances in understanding the molecular pathways of ACP have paved the way for targeted drugs, showing promise for therapy. There is a diversity of treatment models for ACP, and determining the optimal approach remains a subject of ongoing debate in the present context. In order to achieve a good-term quality of life (QOL), the main goal of the cyst disappearance or reduction of surgical treatment is still the main. Additionally, there should be a greater emphasis on personalized treatment at this particular stage and the consideration of ACP as a potentially chronic neurosurgical condition. This review navigates the evolving landscape of ACP therapies, fostering ongoing discussions in this complex field.

## 1 Introduction

ACP, or adamantinomatous craniopharyngioma, is a benign tumor originating from residual epithelial cells of Rathke’s pouch or the craniopharyngeal duct during the embryonic period (1, 2). Approximately 90% of ACP cases manifest as a cystic component (3), with incidence peaks observed in children aged 5 to 15 years and adults aged 45 to 60 years (4, 5). ACP stands as the prevalent non-neuroepithelial intracranial tumor in children, constituting approximately 5–11% of intracranial tumors within the pediatric age cohort (5). Despite being classified as a WHO Grade I tumor, ACP poses a significant challenge in neurosurgical treatment. This complexity arises from its proximity to vital structures like the optic nerve, thalamus, pituitary gland, and the circle of Willis. Additionally, at a histological level, the presence of finger-like protrusions facilitates tumor infiltration and circumferential growth, leading to indistinct boundaries (5–9). This distinctive anatomical and histological context underscores the surgical difficulty posed by ACP. Consequently, these factors contribute to a notable recurrence rate of ACP, persisting even after macroscopic total resection. This recurrence highlights the challenge of achieving complete eradication of the tumor. Even more concerning are the enduring sequelae stemming from surgical resection, encompassing hypothalamic syndrome, severe obesity, diabetes insipidus, visual impairments, and neurocognitive dysfunction (5, 10, 11). These persistent effects lead to a sustained decline in the overall QoL for affected patients.

While the concept of craniopharyngioma (CP) was initially proposed by Cushing in 1929 (12), its precise origin continues to be a subject of debate. Recent studies indicate that β⁃catenin and tumor stem cell markers are predominantly located within the histological finger-like protrusions (FP) (13). This observation suggests a potential association between FP, the origin of ACP, and the invasion of the hypothalamic-pituitary axis by the tumor. Nevertheless, this hypothesis requires additional empirical validation. Consequently, a comprehensive comprehension of ACP’s biology and vigilant monitoring of the latest developments in treatment modalities hold paramount importance in enhancing the management of this highly complex tumor and ameliorating the quality of life for afflicted individuals.

## 2 Materials and methods

Studies included in this article were systematically searched in the PubMed and MEDLINE databases, with the last update conducted in November 2023. Keywords used in the search primarily consisted of “adamantinomatous craniopharyngioma,” “cystic craniopharyngioma,” “craniopharyngioma,” “pediatric” and “childhood-onset” The screening process involved evaluating titles and abstracts, with full texts downloaded for further review if deemed necessary. Additionally, reference lists from extracted studies were thoroughly reviewed. Selection criteria focused on literature concerning advancements in ACP treatment, pathophysiology, tumor control, and patient quality of life, particularly emphasizing recent research, innovative methodologies, and therapeutic strategies. The goal was to provide a comprehensive and contemporary insight into clinical practices by examining the latest developments in ACP treatment and identifying optimal strategies to balance tumor control with quality of life.

## 3 Pathophysiology

### 3.1 Genetics and inflammation in ACP

Molecular studies have demonstrated that CTNNB1 exon 3 mutations, which encode β⁃catenin, are present in 57%-96% of ACP patients (14, 15). Importantly, these mutations are not detected in the adult-onset papillary histological CP subtype (PCP), underscoring their specificity to ACP. This mutation represents the sole known recurrent genetic aberration observed in ACP to date (10). CTNNB1 mutations have the capacity to impede the phosphorylation and degradation of β⁃catenin, resulting in its accumulation within the nucleus. This event triggers the subsequent activation of the WNT/β⁃catenin signal transduction pathway, ultimately culminating in tumorigenesis (16, 17). Studies have demonstrated that CTNNB1 mutations serve as oncogenic drivers in mouse models. Specifically, the expression of a functionally equivalent form of mutant β⁃catenin, akin to that observed in human ACP, in murine SOX2+ embryonic progenitors or adult stem cells leads to the development of tumors (17, 18). Additionally, these mouse models have unveiled novel targets that hold potential therapeutic value in the treatment of ACP (19).

The cystic and solid components of ACP both exhibit a wide array of cytokines, chemokines, and inflammatory mediators. This suggests that they likely share similar molecular characteristics and undergo common molecular events in the course of tumor pathogenesis (20). Elevated levels of cytokines including IL-6, IL-8, CXCL1, and IL-10 have been documented in the cystic fluid of ACP (21). Notably, IL-6 appears to play a role in instigating an inflammatory response in adjacent non-tumor tissues (19, 22). Furthermore, the expression patterns of these cytokines align with the activation of the inflammasome. This response may be initiated by the presence of cholesterol crystals within ACP (5, 23). The notable abundance of α-defensins identified in the cystic fluid implies that inflammation plays a crucial role in prompting the secretion of cyst fluid by epithelial cells lining the cyst wall. Additionally, this finding negates the possibility that the formation of ACP cyst fluid arises from blood-brain barrier disruption. Instead, it suggests that the innate immune response may be implicated in the pathological process of ACP cyst formation (24). These observations provide additional affirmation of the pivotal role played by inflammation in the pathogenesis of ACP.

### 3.2 Morphological and histological features

ACP shares similarities with adamantinoma and post-keratinized odontogenic cysts, typically presenting as calcified, cystic, and lobulated formations (25). From a macroscopic perspective, ACP can manifest as either purely cystic or cystic-solid structures. The solid portions exhibit an amorphous quality, containing numerous micro-calcifications. The cystic fluids are primarily composed of cholesterol crystals and cell fragments, imparting a distinctive “machine oil” appearance (20). The outer layer of the cystic wall comprises fibrous tissue, rendering it resilient and often challenging to puncture. Meanwhile, the inner layer is characterized by an incomplete stratified squamous epithelium, featuring scattered tumor cells extending into the cavity (26).

Histologically, tumors are distinguished by the presence of a peripheral basal cell layer of palisading epithelium, along with the aggregation of loosely arranged stellate cells. The solid components of the tumor typically exhibit distinctive accumulation of “wet” keratin and calcium salts (9, 27). The solid tumors have the propensity to infiltrate surrounding neural tissue in a finger-like manner. Consequently, this aggressive growth pattern can result in severe endocrine and visual dysfunction, as well as substantial surgical morbidity and high recurrence rates, rendering treatment highly challenging (5–9).

Unlike the diverse anatomical classification methods applied to PCP, the cystic nature of ACP facilitates tumor growth in various locations, including the anterior and middle cranial fossae, interpeduncular cisterna, ramus, cerebellar pontine region, and there have even been partial reports of ectopic ACP cases (28–31). Currently, there exists no pertinent literature on the anatomical classification of ACP. To establish a more precise anatomical classification for ACP, it is imperative to accumulate a substantial volume of case data and establish multi-center international registries. These efforts are crucial in providing guidance for clinical imaging identification and treatment approaches.

## 4 Evolution of visual function and endocrine status

Visual impairment in cases of ACP primarily stems from compression of the optic nerve pathway (8, 32, 33). Preoperative assessments have shown that deficits in visual acuity and visual field may reach levels as high as 70-80% (8, 34). The extent of visual impairment is contingent on the lesion’s anatomical positioning in relation to the optic chiasma, with bitemporal hemianopia being a characteristic symptom. Recent studies have demonstrated that, even with straightforward cyst decompression, there can be a substantial improvement in visual function, with a response rate exceeding 75% (32, 35–37). This reaffirms that visual impairment predominantly arises from compression rather than direct tumor invasion. Nevertheless, following craniotomy, the occurrence of postoperative visual impairment may reach levels as high as 30% (38–40). This is primarily attributable to the tumor’s close adherence to surrounding structures and its size (41).

It is imperative to measure hormones perioperatively and during follow-up to mitigate endocrine disorders (42). Preoperatively, the most prevalent endocrine deficiencies encompass growth hormone and gonadotropin deficits. Postoperatively, the most typical endocrine disorders include diabetes insipidus, hypothyroidism, adrenocortical dysfunction, and sexual dysfunction (43–47). The risk of endocrine deterioration is intricately tied to the type of surgical procedure (48–51). Craniotomy poses a higher risk compared to transsphenoidal sinus surgery (52–54), while partial resection carries a lower risk than total or subtotal resection (54). Minimally invasive cyst drainage yields a more favorable endocrine effect compared to surgical resection (31, 35–37, 55). While hormone replacement therapy continues to be the principal approach for addressing endocrine disorders, its administration requires circumspection, and a tailored treatment regimen must be devised (42).

## 5 Surgical strategy

Currently, surgery remains the foremost effective approach for treating ACP. The paramount objective of surgical intervention is to optimize resection safety while preventing irreversible harm to the hypothalamus. Nevertheless, surgical resection of ACP presents two significant challenges. Initially, while complete tumor resection may offer a potential cure for ACP, the recurrence rate remains elevated even after achieving full macroscopic removal. This is primarily due to the tissue structure of the finger-like projections, which can infiltrate and extend into critical neighboring structures like the hypothalamus and pituitary gland. Consequently, this often results in an indistinct demarcation between the tumor and healthy tissue. Attempting a broader resection can frequently lead to severe morbidity (5–9). Secondly, ACP can attain significant size, and not infrequently, its cyst wall becomes intimately attached to vital adjacent structures. Maintaining contact with the exceedingly thin cyst wall during surgery can be challenging, potentially leading to inadvertent detachment. Additionally, the cystic wall may have an ambiguous boundary with the arachnoid interface, further complicating complete resection (56–60). This challenge is particularly pronounced in cases where tumors reach a substantial size, possibly extending into the posterior cranial fossa and other regions. In such instances, employing a combination of approaches may be imperative to achieve complete cyst wall resection. However, this can potentially result in heightened surgical morbidity (61–65). A recent systematic review encompassing 17 studies on adult craniopharyngiomas was conducted (66). It involved 748 patients in the Gross Total Resection (GTR) cohort and 559 patients in the Subtotal Resection (STR) cohort. The findings indicated that GTR significantly reduced the likelihood of recurrence (OR, 0.106; 95% CI, 0.067-0.168; P < 0.001), albeit at the expense of an increased occurrence of postoperative panhypopituitarism (OR, 2.063; P = 0.034) and permanent diabetes insipidus (OR, 2.776; P = 0.007).

## 6 Endonasal endoscopic surgery

Over the last two decades, Endonasal Endoscopic Surgery (EES) has seen a growing utilization in the treatment of ACP, owing to its benefits of microinvasion and enhanced visualization (67–69). The 2020 EANS consensus statement on adult CP treatment advises utilizing the endonasal approach for midline CP, whereas transcranial approaches are suggested for lateral extension or purely intraventricular growth tumors (70). A recent systematic review comparing transcranial endoscopic and transcranial approaches to craniopharyngioma excision demonstrated that EES excision yielded a higher total excision rate and a greater likelihood of vision improvement (61.3% vs. 50.5%) in eight studies encompassing 376 patients. In cases where both approaches achieve complete tumor removal, EES exhibits superiority over TCA regarding GTR rate and visual outcomes. Additionally, it demonstrates positive effects on complications aside from cerebrospinal fluid (CSF) leakage, including hypopituitarism and diabetes insipidus (71). CSF leakage is a relatively prevalent complication following EES. However, this incidence is on a decreasing trend with the maturation of surgical proficiency and advancements in skull base repair techniques (71). In 2023, Qiao et al. conducted a comprehensive analysis of 364 craniopharyngioma patients who underwent EES over a span of ten years (72), representing the largest series to date. The study identified a larger dural defect size (OR 8.545, 95% CI 3.684-19.821, p < 0.001) and lower preoperative serum albumin levels (OR 0.787, 95% CI 0.673 to 0.919, p = 0.002) as independent risk factors for postoperative CSF leakage. Interestingly, CSF leakage was not associated with the opening of the third ventricle floor.

**Figure 1:**
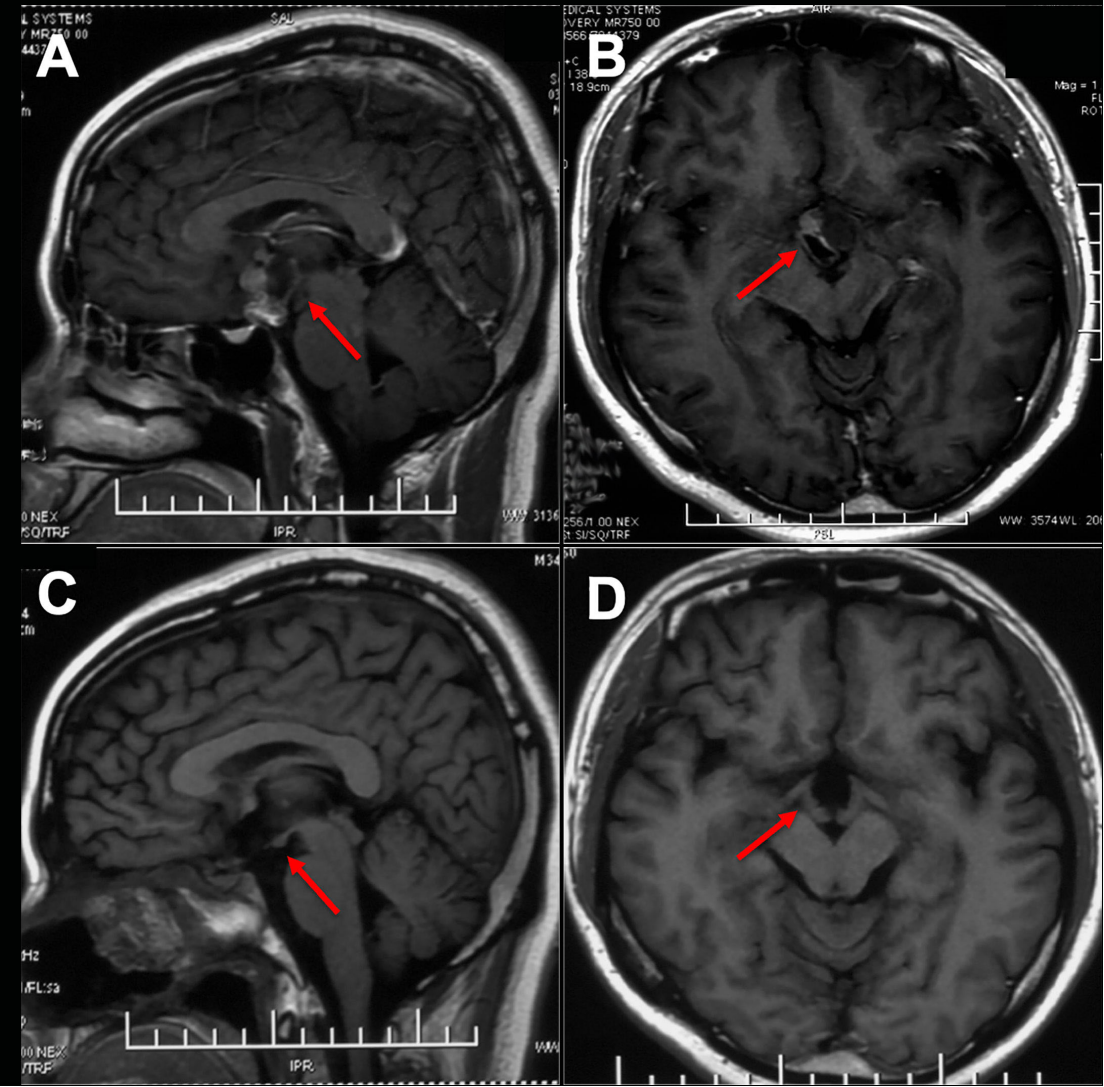
Preoperative and postoperative imaging of an intrinsic third ventricle craniopharyngioma with an intact mammillary body was documented ([aken reference from Zhou et al. (74)]. In images **(A, B)**, a cystic and solid lesion was observed in the suprasellar region, while postoperative MR images (**C, D)** displayed the intactness of the optic tract, third ventricle floor, and mammillary bodies.

**Figure 2:**
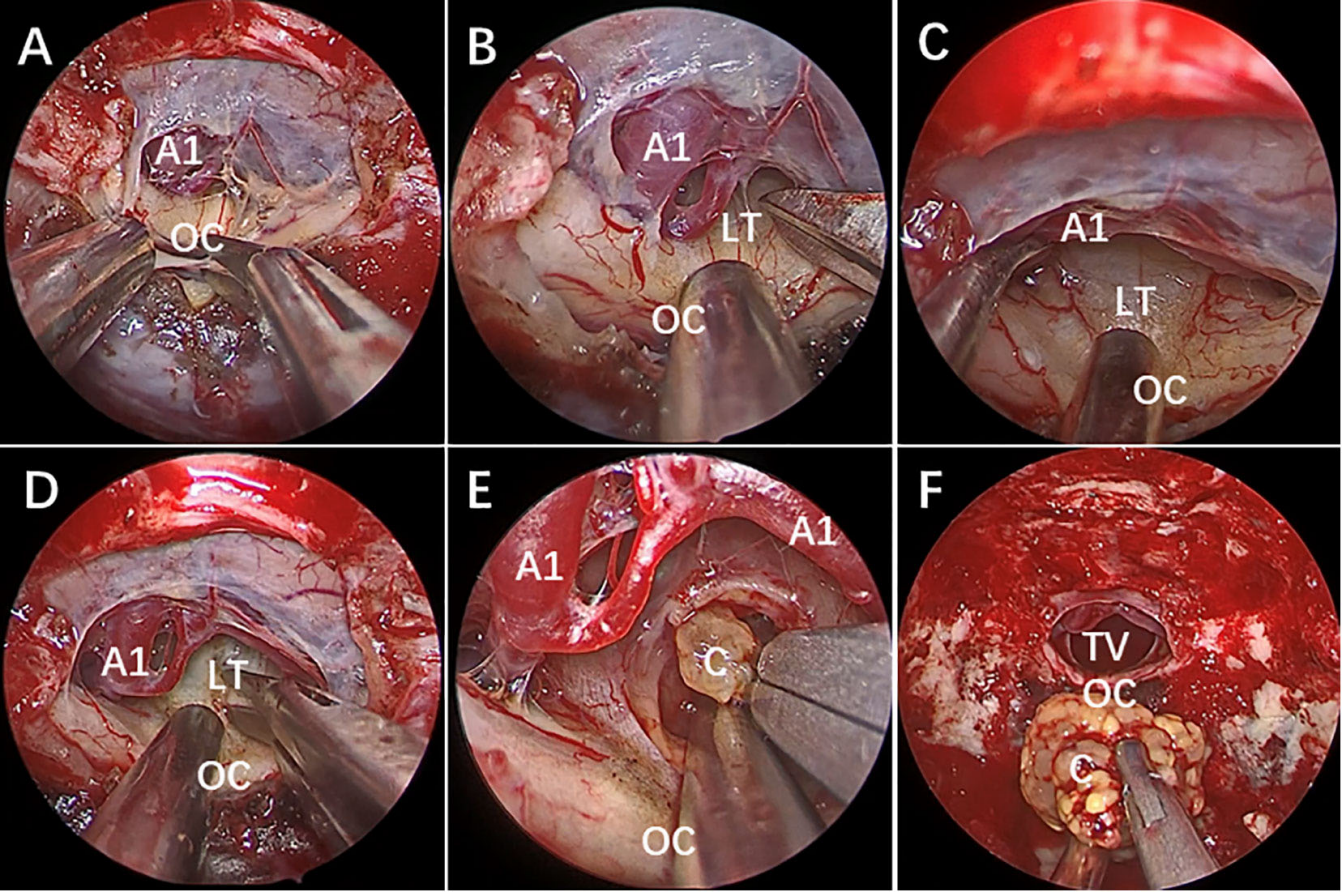
Endoscopic endonasal surgery (EES) was performed to remove the third ventricle Craniopharyngioma by STLT approach. [taken reference from Zhou et al. (74)]. **(A)** The optic chiasm exposure after the dura mater opening. **(B)** Lamina terminalis exposure after anterior longitudinal division and anterior circulation artery arachnoid membrane dissection. **(C)** Pulling the optic chiasm downward and the anterior circulation artery system upward to fully exposed the lamina terminalis and the surgical approach, and no tumor was found in the narrow infrachiasmatic space. **(D, E)** The anterior part of the third ventricle tumor was exposed after lamina terminalis incision, and the tumor was debulked and removed using suction tube and grasping forcep piece by piece; **(F)** Complete removal the third ventricle tumor without surgical-related injury. OC, optic chiasm; A1, A1 segment of anterior cerebral artery; LT, lamina terminalis; TV, third ventricle; C, craniopharyngioma.

While Endonasal Endoscopic Surgery (EES) provides excellent visual field exposure to the subchiasmatic, postchiasmatic, and pituitary stalk-infundibular axes, its effectiveness is limited in cases of third ventricle ACP, particularly when the tumor extends bilaterally into the thalamus, leading to significant vaso-neurostructural lesions in its vicinity (73–75). Cavallo et al. (73) reported on a cohort of 103 patients with Craniopharyngioma, achieving a GTR rate of 68.9%. However, this rate decreased to 30% when the lesions extended bilaterally into the thalamus. In their another study (76), EES was employed to treat 13 patients primarily presenting with third ventricle ACP. Among them, GTR was achieved in 8 patients (66.7%). However, two patients experienced subdural hematomas, and one patient tragically succumbed to brain stem hemorrhage. Zhou et al. reported the utilization of EES in treating 14 patients with intrinsic third ventricle craniopharyngioma using the Suprachiasmatic Endplate (STLT) approach, 13 (92.8%) attained GTR, while the remaining patient achieved near-total resection (90%). Additionally, 3 patients necessitated hormone replacement therapy, and 1 patient experienced a decline in vision. The average follow-up period was 26.2 months, during which no instances of tumor recurrence were noted (74). While long-term follow-up data is lacking, their experience indicates that accessing the endplate offers superior exposure to the third ventricle environment and reduced surgery-related injury. This may present a novel neuroendoscopic option for treating third ventricle ACP (**Figure 1**, **Figure 2**).

## 7 Management of cyst (as a chronic neurosurgical disease)

### 7.1 Neuroendoscopic fenestration

Due to the benefits of endoscopic visualization, some earlier studies reported the safety and feasibility of employing endoscopic transventricular treatment for ACP. Over the past five years, a number of relatively small-scale series have serendipitously discovered that endoscopic fenestration for ACP has led to low surgical morbidity and a relatively extended period of disease control. Lauretti et al. reported that they achieved enduring tumor control by utilizing endoscopy to create a large opening in the upper section of the cyst and they attributed this mainly to the establishment of a permanent connection between the cyst cavity and the CSF space. Among the eight patients, there was a recurrence rate of 20% and a median progression-free survival (PFS) of 57 months. The authors identified endoscopy as an independent predictor of reduced cyst recurrence (37). Hollon et al. employed the “through-and-through” technique, conducting wide cyst fenestration at the top and bottom of the cyst for cystic retrochiasmatic craniopharyngiomas. Some patients received postoperative radiotherapy. The study had a mean follow-up of 2.5 ± 1.6 years, and a noteworthy decrease in postoperative cyst volume was noted. The patients experienced substantial enhancement in their quality of life, and there were no surgical complications (35). In a similar vein, Takano et al. accomplished long-term tumor control in 8 out of 9 patients (88.9%) over a mean follow-up of 72.9 months. This was achieved through fenestration and irrigation solely at the upper portion of the cyst, followed by segmental stereotactic radiotherapy. This led to significant alleviation of symptoms like cranial hypertension and visual impairment. No surgical complications arose (36).

### 7.2 Stereotactic cyst drainage

Steiert et al. conducted stereotactic-guided catheter implantation in 12 cases of cystic craniopharyngioma. Over an average follow-up period of 41 months, the cyst volume reduced by 64.2%. Additionally, post-radiotherapy, there was an average reduction of cyst volume by 92.0%. They also believe that establishing connection between the cyst cavity and the CSF space is an important factor in preventing the cyst reaccumulate. Furthermore, the patients experienced a significant improvement in visual function without incurring any new complications (77). Rachinger et al. conducted microsurgery and implemented stereotaxic “bidirectional drainage” in 79 cases of craniopharyngiomas. The median follow-up duration was 51 months (range: 14-188 months). The findings revealed that while the PFS for cystic tumors was relatively brief (5-year PFS: 53.6% vs 66.8%, p = 0.10), bidirectional drainage yielded significantly improved endocrine outcomes. The authors suggested that a majority of patients with cystic craniopharyngioma might not necessitate early radiotherapy post-drainage (32).

**Figure 3:**
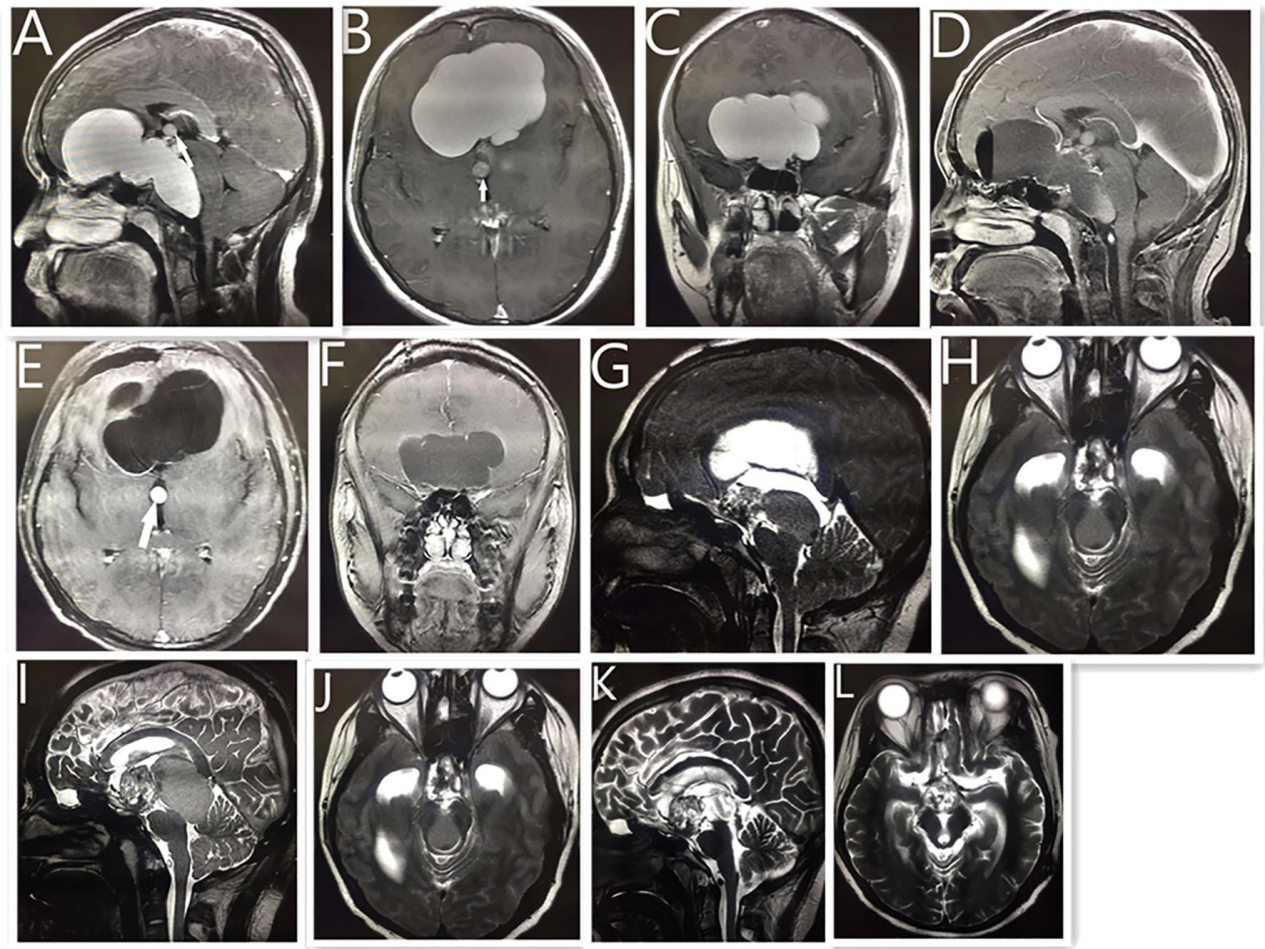
Neuroendoscopic treatment of giant cystic craniopharyngioma in a 15-year-old boy. MRI **(A–C)** showing a giant cystic lesion on the front middle, and posterior fossa. White arrow shows small cystic tumor of the third ventricle. Postoperative MRI reveals no obvious cystic tumor after neuroendoscopic surgery **(D–F)**. Five years later, the MRI reveals the previous tumor had disappeared, with a solid lesion on the suprasellar region **(G)** and a cystic lesion compressing the mid brain aqueduct **(H)**, which caused hydrocephalus. The patient received a ventriculoperitoneal shunt, and his condition improved. One year later, MRI reveals **(I, J)** that the tumor had become larger than before. Postoperative MRI reveals that **(K, L)** the cystic lesion disappeared and the midbrain aqueduct opened up after the neuroendoscopic surgery. As of 2 years into follow-up, the size of the remaining tumor had not changed, and the patient had continued to live a normal life.

Given the surgical resection’s associated morbidity and the high recurrence rate of ACP post-operation, the amassed clinical data are progressively highlighting the constraints of traditional surgical approaches. Lately, there has been a growing interest in the management of cysts via tumor-focused methods (**Table 1**). This is primarily due to the potentially lower incidence rate compared to alternative surgical procedures, allowing patients to attain a satisfactory quality of life, particularly those with hydrocephalus (35–37, 75, 77). This staged treatment approach may be more strongly recommended. While the present treatment of ACP through cyst drainage, either independently or in conjunction with other modalities, recently observed clinical outcomes seems to have brought about notable improvements, especially in the patients’ quality of life, but there are still some problems several problems. Firstly, the enduring effects on ACP may still be constrained, whether achieved through endoscopic cyst fenestration or basic drainage. The aftermath of these procedures is that cysts are prone to reaccumulate, solid tumors grow, and more importantly, recurrent tumors may complicate the initial treatment. Secondly, in theory, establishing a connection between the cyst cavity and the CSF space to use the CSF flow to reduce cyst reaccumulate may be an ideal way, but no studies have confirmed the exact effectiveness of this approach and it is uncertain whether the enlarged cyst opening will close. Thirdly, given the visual benefits of endoscopy, cysts can potentially be treated more comprehensively under direct visualization (**Figure 3**). Nevertheless, the extent to which endoscopy can conclusively be identified as a potential factor in reducing the recurrence rate of ACP remains uncertain, and there is still a lack of standardized guidelines for endoscopic therapy techniques. Additionally, there exists a diversity in the techniques for cyst drainage. The likelihood of cyst reformation and the safety of the procedure have not been thoroughly compared across different surgical approaches.

**Table 1:** Summary of Cyst Drainage for Cystic Craniopharyngiomas.

Study	Number of cases	Morphology	Drainage method	Radiotherapy	Follow-Up (months)	Recurrence Rate	Complications
Delitala et al., 2004 (78)	7	Cystic	Endoscopy	No	38	28%	No
Tirakotai et al., 2004 (79)	10	Mixed	Endoscopy	No	NA	20% (solid)	No
Schubert et al., 2009 (80)	7	Cystic	Stereotaxy	Yes	118	29%	No
Park et al., 2011 (48)	13	Cystic	Endoscopy	Yes	32	54%	No
Moussa et al., 2012 (81)	52	Cystic	Stereotaxy	No	54	27%	No
Takano et al., 2015 (36)	9	Cystic	Endoscopy	Yes	73	11%	No
Rachinger et al, 2016 (32)	31	Cystic	Stereotaxy	No	51	42%	Intratumo al bleeding (1)
Lauretti et al., 2017 (37)	8	Cystic	Endoscopy	No	56	12.5%	CSF leakage
Frio et al. 2019 (82)	11	Cystic	Endoscopy+ Stereotaxy	No	41.4	27.3%	CSF leakage
Hollon et al. 2017 (35)	10	Cystic	Endoscopy	Yes	30	10% (solid)	No
Steiert et al. 2021 (77)	12	Cystic	Stereotaxy	Yes	41	0	Dislocation of the catheter
Chen et al. 2022 (55)	15	Cystic	Endoscopy	No	67	33%	No

CSF, cerebrospinal fluid.

The limitations of the present literature on cyst management of ACP predominantly arise from the varying definitions of cystic recurrence in existing studies, making comparisons between different research endeavors challenging. Additionally, these clinical data primarily originate from patients treated in varying selective settings, often lacking extensive long-term follow-up (22), but these clinical experiences may open up a new potential avenue for the treatment of ACP.

### 7.3 Intracystic catheter and reservoir system

Given the potential reaggregation of cysts and the emergence of new clinical symptoms due to cystic occupation, an intracystic catheter and reservoir (such as a Rickham or Ommaya reservoir) are considered for implantation. In the majority of cases, it is feasible to establish a sustainable decompression regimen to address cyst progression (22, 82). Mouss et al. documented 52 patients with cystic craniopharyngioma, and with a minimum follow-up of 7 years, they were astounded to discover that 38 patients (73%) experienced no recurrence of cysts and necessitated no further treatment (81). Various methods for catheter implantation in the capsular cavity exist, encompassing neuroendoscopy, stereotactic techniques, neuronavigation, freehand placement, and intraoperative MRI, among others (81–85). The prevailing trend in development is the emphasis on precision and visualization in catheter placement. Nevertheless, catheter placement may give rise to infrequent complications like infection, bleeding, CSF leakage, catheter obstruction and displacement (32, 81, 82, 86–88). Lau et al. (87) undertook a systematic review of 43 studies and determined that the use of image guidance during implantation resulted in fewer complications compared to procedures without it. In our experience, it is challenging to completely aspirate the cyst contents during the initial surgery and accurately gauge the extent of cyst component removal in the absence of a clear visual field. It is important to note that the high viscosity of the “oil-like” cystic fluid, along with substantial calcification, can hinder later aspiration efforts, potentially necessitating catheter replacement after obstruction. Furthermore, even with precise preoperative positioning, certain rigid or elastic cyst walls may diminish the puncture fault tolerance rate (89), and forceful puncture may result in cystic bleeding (32, 48). However, visualization techniques like endoscopy can mitigate these issues, particularly in the case of polycystic or large craniopharyngiomas that extend into the posterior fossa. Employing a reservoir establishes a secure conduit for intracapsular irradiation and heightens sensitivity to subsequent radiation therapy by facilitating the removal of more fluid.

## 8 Radiotherapy

Radiotherapy serves as a crucial adjuvant therapy for ACP. Extensive clinical data and meta-analyses indicate that subtotal resection with radiotherapy (STR+RT) may yield tumor control rates similar to, or even superior to, those achieved by GTR, particularly in terms of endocrine outcomes (90–92). The 5-year PFS rates were 67-77% for GTR and 69-73% for STR in combination with radiotherapy (93, 94). Importantly, STR+RT did not elevate the risk of long-term onset or central diabetes insipidus. Notably, there is no substantial disparity in PFS across various radiological techniques (22). Proton therapy offers benefits over photon irradiation by minimizing the dosage received by closely situated critical structures, potentially ameliorating both acute and long-term toxic effects of radiation therapy. Findings from a recent single-arm Phase II clinical trial (NCT01419067) evaluating proton therapy in conjunction with limited surgery for craniopharyngioma (RT2CR) in children and adolescents demonstrated that proton therapy led to superior cognitive outcomes compared to photon therapy (95). However, no significant differences were observed in other aspects. However, there are several concerns associated with radiotherapy for ACP: (1) The ideal timing for radiotherapy remains uncertain—whether as an early adjuvant measure or as a salvage intervention. Limited-quality clinical evidence indicates that salvage radiotherapy may elevate the risk of visual and endocrine complications, although it does not confer a survival advantage. (2) Cyst growth is frequently an unpredictable phenomenon (61, 96, 97). Cysts can exhibit either rapid or gradual expansion at any given time, introducing instability in the potential coverage of the target region. Hence, periodic imaging throughout radiotherapy may be imperative to ascertain precise coverage. (3) Given the relative resistance of cysts to radiotherapy, surgical intervention or cyst drainage is often required in most cases to diminish the overall cyst volume, thereby enhancing the efficacy of radiotherapy (36, 48, 86). (4) In cases of mixed cystic and solid tumors, it is possible that solid and cystic components exhibit distinct responses to radiotherapy (98–100). Given the potential autonomy of these components, some studies have explored the combination of stereotactic radiotherapy (SRS) and endovascular radiotherapy as a therapeutic strategy for mixed ACP (101).

## 9 Intracavitary treatment

### 9.1 Intracavitary brachytherapy

Intracavitary Brachytherapy (IBT) has demonstrated effectiveness in ACP treatment while minimizing radiation exposure to adjacent normal tissue structures. A recent review (102) illustrated the favorable impact of IBT on cystic craniopharyngiomas. Among 228 patients with purely cystic lesions, 89% exhibited either complete or partial responses. This intervention led to visual and endocrine enhancements of 64% and 20%, respectively. In contrast, mixed cystic tumors showed less favorable outcomes. However, in the most extensive cohort (90 patients) with the lengthiest follow-up period (mean 121 months, ranging from 60 to 192 months), individual ACP patients treated with P32 brachyluminal irradiation exhibited significant results. Specifically, 56 cysts (43.4%) experienced complete regression or remained recurrence-free, while 47 cysts (36.4%) demonstrated a partial response. Five cysts (3.9%) remained stable. Additionally, complications arose in 7 patients (7.8%) (103). In two other extensive series (104, 105) encompassing 53 and 49 patients respectively, 5-year tumor control rates were 86% and 76%. Complications were observed in 5.9% and 6.1% of cases. The aforementioned comprehensive series of studies have demonstrated that IBT stands as a dependable method for treating ACP in the short to medium-term follow-up (**Table 2**). However, complications occur primarily stemming from radiation-induced damage due to nuclide leakage. Another constraint lies in IBT’s reduced efficacy against the solid component of mixed tumors. This elucidates why IBT proves more efficacious in managing purely cystic tumors as opposed to mixed ones.

**Table 2:** Review of the literature on Phosphorus-32 use in the treatment of cystic craniopharyngiomas.

Study	NO. of patients	Mean Age (years)	Radiation dose (mean)	Previous treatments	Response	Follow-Up (months)	Complications (N)
POLLACK, et al. 1988 (106)	9	–	200-300Gy	Surgery (22.2%)	Complete (22.2%) Partial (77.8%)	27	None
POLLOCK, et al., 1995 (107)	30	26	253Gy	Surgery (50%) RT (33.3%)	Complete (10%) Partial (83.3%) Stable (3%) Progression (20%)	37	VA (2), DI (2), AB (3)
Shahzadi et al. 2008 (108)	22	14	250Gy	Surgery (95.4%) RT (45.5%)	Complete (27.2%) Partial (45.5%) Stable (18.2%)	10.5	None
Zhao et al., 2009 (60)	20	6.2	400-500Gy	Surgery (58.8%)	Complete (30%) Partial (70%)	47.7	None
Barriger et al., 2011 (109)	19	20	290.5Gy	Surgery (63.2%) RT (5%)	Complete (5%) Partial (26.3%) Stable (10.5%) Progression (57.9%)	62	None
Hasegawa et al., 2011 (104)	41	29	224Gy	Surgery (52.8%) RT (24%)	Complete (17%) Partial (58.5 %) Stable (12.2 %) Progression (12.2%)	60	VA (3) DI (3)
Yu et al., 2014 (110)	20	6.7	150Gy	Surgery (15%) RT (5%)	Complete (60%) Partial (40%)	48.6	None
Maarouf, et al, 2015 (111)	17	15.4	200Gy	Surgery (58.8%) RT (17.6%)	Complete (5%) Partial (29.4%) Stable (17.6%) Progression (17.6%)	61.9	None
Kickingerer, et al., 2021 (105)	53	31.1	200Gy	Surgery (28) RT (18.9%)	Complete (29.4 %) Partial (52.9 %) Stable (5.9 %) Progression (11.8 %)	60.2	Hemiparesis (1) TNP (1)
Yu et al., 2021 (103)	90	36.6	250Gy	Surgery (61%) RT (15.6%)	Complete (43.4%) Partial (36.4%) Stable (3.9%)	121	VA (4), TNP (2), CAO (1)

VA, Visual abnormalities; TNP, Third nerve palsy; CAO, Carotid artery occlusion; DI, Diabetes insipidus; AB, Abnormal behaviour.

### 9.2 Intracavitary chemotherapeutic treatment

An alternative approach to enhance tumor control while minimizing radiation exposure involves exploring alternative intracapsular injection substances, including bleomycin and interferon. In several extensive retrospective studies employing bleomycin (112–114), Follow-up times ranged from 2 to 10 years. patients exhibited complete cyst disappearance rates ranging from 29% to 67%. Most cysts experienced varying degrees of reduction. Nonetheless, patients often experience complications such as headaches, nausea, and vomiting following administration. Simultaneously, nuclide leakage can potentially inflict damage on the hypothalamus and optic nerve, and in severe cases, lead to fatality (115–117). The clinical evidence from three consecutive reviews (118–120) does not favor the utilization of bleomycin in ACP, given the trade-off between benefits and complications. It is recommended that randomized controlled trials employing standardized dosing regimens be conducted to ascertain the safety and efficacy of bleomycin in tumor treatment. In contrast to bleomycin, which is linked to more prevalent adverse reactions in ACP treatment, a recent systematic review of intracapsular drugs for ACP indicated that intratumoral interferon alpha appeared to yield the most favorable response with minimal side effects in ACP treatment when compared to other drugs (121). Cavalheiro et al. conducted a prospective multi-center analysis involving 60 cases of ACP. Clinical and radiological improvements were observed in 76% of cases, with a small subset of patients experiencing minor side effects like mild headaches and eyelid edema (122). Kilday (123) et al. conducted a clinical trial involving 56 children from 21 international centers. Among them, 43 (77%) patients had received other treatments prior to interferon. Intracapsular interferon was found to impede further tumor progression compared to previous treatments. Following interferon therapy, 42 patients experienced progression (median time of 14 months; range of 0-8 years). The estimated median time to reach the final treatment after interferon therapy was 5.8 years (ranging from 1.8 to 9.7 years), and significant side effects were infrequent. However, the recently published National UK guidelines for the management of pediatric craniopharyngioma do not provide adequate evidence to endorse IFNα as the preferred first-line treatment option. Additionally, intracystic bleomycin and radioisotopes lack robust support as the primary strategies for ACP treatment (124).

## 10 Target therapies

As our understanding of ACP’s pathogenesis advances, there is a growing optimism regarding the clinical application of targeted drugs aimed at the growth and molecular pathways associated with ACP. MEK inhibitors have demonstrated the ability to decrease tumor cell count, inhibit cell proliferation, and induce apoptosis by impeding the MAPK/ERK signal transduction pathways Park (125). In a compassionate treatment approach, a 26-year-old woman, previously subjected to multiple surgeries, received binimetinib for 8 months, resulting in a reduction in tumor volume. Although the reduction was not notably substantial, this case presents additional avenues for MEK as a potential target in ACP treatment (126). Simultaneously, drugs targeting IL-6 appear to elicit a more substantial reduction in volume during ACP treatment (127). Two patients with recurrent ACP experienced noteworthy reductions in tumor volume after receiving either tocilizumab or a combination of tocilizumab and bevacizumab. At present, the targeted therapy for the above targets has shown an optimistic attitude in the treatment of ACP for the time being, but further clinical and basic research is still needed to determine their specific efficacy in the treatment of ACP.

## 11 Childhood-onset craniopharyngioma

Childhood-onset craniopharyngioma predominantly manifests as Adamantinomatous Craniopharyngioma (ACP), showing a combination of cystic, solid, and calcified components (128). In contrast to the adult CP, its diagnosis often occurs late, featuring clinical indications like increased intracranial pressure, alongside endocrine deficits and visual impairment (5). Therapeutic strategies for childhood-onset CP encompass surgical resection, radiotherapy, cyst aspiration, and intracavitary chemotherapy. However, recent guidelines lack a clearly defined optimal treatment plan (124). Surgical outcome is often associated with a high recurrence rate. A comprehensive review (93) involving 109 studies and 532 cases of childhood-onset CP undergoing surgical resection revealed recurrences in 377 cases. The 5-year progression-free survival for GTR, STR+XRT, and STR alone stood at 77%, 73%, and 43%, respectively. GTR and STR+XRT exhibit similar tumor control rates, with GTR demonstrating relatively superior tumor control effects compared to STR alone. However, adjuvant radiotherapy is often employed as a salvage measure.

In the past, the use of the Endoscopic Endonasal Approach (EEA) in pediatric Craniopharyngioma (CP) appeared controversial due to unique considerations in children’s nasal anatomy and the risk of CSF leakage. However, a recent systematic review of pediatric CP seems to challenge this perception (129). This review encompassed 835 patients underwent TCA (18 articles) and 403 patients underwent EEA (19 articles), indicating a rising preference for EEA in pediatric CP, showing favorable outcomes. Analysis from the study showed EEA as the preferred approach (p = 0.006, PI = 26.8-70.8, I2 = 40%) for sellar-suprasellar CPs, while TCA was favored for purely suprasellar CPs (p = 0.007, PI = 13.5-81.1, I2 = 61%). However, no significant difference was observed between the approaches for purely intrasellar lesions (p = 0.94, PI = 0-62.7, I2 = 26%).

Recent two single-center retrospective studies further highlighted the feasibility and safety of EEA in treating pediatric midline CP (130, 131). In a cohort of 25 patients, a GTR reached 92%, with a tumor recurrence rate of 19% over a mean follow-up of 72 ± 67 months, and panhypopituitarism was the most common complication (92%) (130). Another study comparing EEA (35 patients) and TCA (16 patients) in pediatric midline CP found comparable tumor control and surgical complication rates between the approaches (131). Additionally, EEA may be associated with better visual and endocrine outcomes. This shifting preference underscores the increasing role and acceptance of EEA in managing pediatric CP.

While intracavitary chemotherapy or cyst aspiration generally show greater efficacy than conservative approaches, their PFS have not been well described compared to surgical resection in existing literature. In cases of childhood-onset CP accompanied by hydrocephalus, a staged approach involving minimally invasive cyst decompression followed by cyst aspiration or tumor resection is recommended to mitigate clinical risks and achieve effective tumor control (124).

CP invasion into the hypothalamus or surgical injury often leads to the development of hypothalamic syndrome, encompassing hypothalamic obesity and neuropsychological deficits (132). For patients with definite hypothalamic involvement, STR combined with postoperative radiotherapy is recommended to reduce the incidence of long-term obesity without increasing the recurrence rate (133). Accurate preoperative grading of hypothalamic involvement stands pivotal in shaping surgical strategies and preventing hypothalamic injury. Several different hypothalamic grading systems have been developed in the past (19), including recent applications of machine learning in predicting ACP invasiveness through radiomic methodologies (134). Advancements in imaging modalities, such as 7-T MRI or fMRI, significantly enhance hypothalamic structure visualization, aiding neurosurgeons in precise surgical resection. Despite recent enhancements in understanding hypothalamic syndrome, effective pharmaceutical interventions for hypothalamic obesity remain elusive, and data on treating neurocognitive deficits in childhood-onset CP are insufficient (5). Consequently, an effective remedy for hypothalamic syndrome, posing a significant challenge in childhood-onset CP treatment, remains absent. Future research should prioritize exploring the molecular mechanisms underlying CP invasion of the hypothalamus to develop strategies for preventing and treating hypothalamic syndrome.

## 12 Conclusion

Adamantomatous craniopharyngioma (ACP) represents a complex intracranial tumor. Despite significant strides in ACP research and treatment over the past three decades, achieving full control of this often-termed “most challenging brain tumor” remains elusive. Recent clinical perspectives have shifted towards managing cysts and treating ACP as a chronic neurosurgical condition, emphasizing the importance of achieving a high-quality, long-term life for patients. This may open up a new potential avenue for ACP treatment. However, the long-term risks of cyst reaggregation and tumor recurrence still need to be fully evaluated. The primary objective in ACP treatment remains the amalgamation of maximal safe resection with radiation therapy, ongoing cyst decompression, and pharmacotherapy to balance long-term tumor control and quality of life. Simultaneously, owing to ACP’s relative rarity and the tumor’s heterogeneity, conducting clinical and foundational trials to bolster international collaboration across diverse nations will significantly enhance our comprehensive comprehension of this condition.

## Author contributions

AC: Writing – original draft, Writing – review & editing, Conceptualization, Data curation. MA: Data curation. TS: Resources, Supervision.

## References

[1] Goldberg GM Eshbaugh DE. Squamous cell nests of the pituitary gland as related to the origin of craniopharyngiomas. A study of their presence in the newborn and infants up to age four. Arch Pathol (1960) 70:293–9. 13850582

[2] Karavitaki N Cudlip S Adams CB Wass JA. Craniopharyngiomas. Endocrine Rev (2006) 27(4):371–97. doi: 10.1210/er.2006-0002 16543382

[3] Johnson LN Hepler RS Yee RD Frazee JG Simons KB. Magnetic resonance imaging of craniopharyngioma. Am J Ophthalmol (1986) 102(2):242–4. doi: 10.1016/0002-9394(86)90152-2 3740186

[4] Nielsen EH Feldt-Rasmussen U Poulsgaard L Kristensen LO Astrup J Jørgensen JO. Incidence of craniopharyngioma in Denmark (n = 189) and estimated world incidence of craniopharyngioma in children and adults. J neuro-oncology (2011) 104(3):755–63. doi: 10.1007/s11060-011-0540-6 21336771

[5] Müller HL Merchant TE Warmuth-Metz M Martinez-Barbera JP Puget S. Craniopharyngioma. Nat Rev Dis Primers (2019) 5(1):75. doi: 10.1038/s41572-019-0125-9 31699993

[6] Weiner HL Wisoff JH Rosenberg ME Kupersmith MJ Cohen H Zagzag D. Craniopharyngiomas: a clinicopathological analysis of factors predictive of recurrence and functional outcome. Neurosurgery (1994) 35(6):1001–11. doi: 10.1227/00006123-199412000-00001 7885544

[7] Martinez-Barbera JP Buslei R. Adamantinomatous craniopharyngioma: pathology, molecular genetics and mouse models. J Pediatr Endocrinol Metab JPEM (2015) 28(1-2):7–17. doi: 10.1515/jpem-2014-0442 25503464

[8] Drapeau A Walz PC Eide JG Rugino AJ Shaikhouni A Mohyeldin A. Pediatric craniopharyngioma. Child’s nervous system (2019) 35(11):2133–2145. doi: 10.1007/s00381-019-04300-2 31385085

[9] Bi WL Santagata S. Skull base tumors: neuropathology and clinical implications. Neurosurgery (2022) 90(3):243–61. doi: 10.1093/neuros/nyab209 34164689

[10] Hengartner AC Prince E Vijmasi T Hankinson TC. Adamantinomatous craniopharyngioma: moving toward targeted therapies. Neurosurgical Focus (2020) 48(1):E7. doi: 10.3171/2019.10.FOCUS19705 31896087

[11] Henderson J.R. F Schwartz TH. Update on management of craniopharyngiomas. J neuro-oncology (2022) 156(1):97–108. doi: 10.1007/s11060-021-03906-4 34807341

[12] Pascual JM Rosdolsky M Prieto R Winter E Ulrich W. Jakob Erdheim (1874-1937): father of hypophyseal-duct tumors (craniopharyngiomas). Virchows Archiv an Int J Pathol (2015) 467(4):459–69. doi: 10.1007/s00428-015-1798-4 26089144

[13] Li S Wu B Xiao Y Wu J Yang L Yang C. Exploring the pathological relationships between adamantinomatous craniopharyngioma and contiguous structures with tumor origin. J neuro-oncology (2022) 159(2):485–97. doi: 10.1007/s11060-022-04084-7 35939144

[14] Campanini ML Colli LM Paixao BM Cabral TP Amaral FC MaChado HR. CTNNB1 gene mutations, pituitary transcription factors, and MicroRNA expression involvement in the pathogenesis of adamantinomatous craniopharyngiomas. Hormones Cancer (2010) 1(4):187–96. doi: 10.1007/s12672-010-0041-7 PMC10358030 21761366

[15] Semba S Han SY Ikeda H Horii A. Frequent nuclear accumulation of beta-catenin in pituitary adenoma. Cancer (2001) 91(1):42–8. doi: 10.1002/1097-0142(20010101)91:1<42::aid-cncr6>3.0.co;2-7 11148558

[16] Hölsken A Buchfelder M Fahlbusch R Blümcke I Buslei R. Tumour cell migration in adamantinomatous craniopharyngiomas is promoted by activated Wnt-signalling. Acta neuropathologica (2010) 119(5):631–9. doi: 10.1007/s00401-010-0642-9 20131060

[17] Gaston-Massuet C Andoniadou CL Signore M Jayakody SA Charolidi N Kyeyune R. Increased Wingless (Wnt) signaling in pituitary progenitor/stem cells gives rise to pituitary tumors in mice and humans. Proc Natl Acad Sci United States America (2011) 108(28):11482–7. doi: 10.1073/pnas.1101553108 PMC3136310 21636786

[18] Andoniadou CL Matsushima D Mousavy Gharavy SN Signore M Mackintosh AI Schaeffer M. Sox2(+) stem/progenitor cells in the adult mouse pituitary support organ homeostasis and have tumor-inducing potential. Cell Stem Cell (2013) 13(4):433–45. doi: 10.1016/j.stem.2013.07.004 24094324

[19] Apps JR Muller HL Hankinson TC Yock TI Martinez-Barbera JP. Contemporary biological insights and clinical management of craniopharyngioma. Endocrine Rev (2023) 44(3):518–38. doi: 10.1210/endrev/bnac035 36574377

[20] Desiderio C Rossetti DV Castagnola M Massimi L Tamburrini G. Adamantinomatous craniopharyngioma: advances in proteomic research. Child's nervous system (2021) 37(3):789–97. doi: 10.1007/s00381-020-04750-z 32617710

[21] Donson AM Apps J Griesinger AM Amani V Witt DA Anderson RCE Advancing Treatment for Pediatric Craniopharyngioma Consortium. Molecular analyses reveal inflammatory mediators in the solid component and cyst fluid of human adamantinomatous craniopharyngioma. J neuropathology Exp Neurol (2017) 76(9):779–88. doi: 10.1093/jnen/nlx061 PMC6005018 28859336

[22] Bianchi F Benato A Massimi L. Treatment of cystic craniopharyngiomas: an update. Adv Tech standards Neurosurg (2022) 45:139–76. doi: 10.1007/978-3-030-99166-1_4 35976449

[23] Apps JR Carreno G Gonzalez-Meljem JM Haston S Guiho R Cooper JE. Tumour compartment transcriptomics demonstrates the activation of inflammatory and odontogenic programmes in human adamantinomatous craniopharyngioma and identifies the MAPK/ERK pathway as a novel therapeutic target. Acta neuropathologica (2018) 135(5):757–77. doi: 10.1007/s00401-018-1830-2 PMC5904225 29541918

[24] Pettorini BL Inzitari R Massimi L Tamburrini G Caldarelli M Fanali C. The role of inflammation in the genesis of the cystic component of craniopharyngiomas. Child's nervous system ChNS Off J Int Soc Pediatr Neurosurg (2010) 26(12):1779–84. doi: 10.1007/s00381-010-1245-4 20668862

[25] Rushing EJ Wesseling P. Towards an integrated morphological and molecular WHO diagnosis of central nervous system tumors: a paradigm shift. Curr Opin Neurol (2015) 28(6):628–32. doi: 10.1097/WCO.0000000000000258 26402407

[26] Okada T Fujitsu K Ichikawa T Miyahara K Tanino S Niino H. Unicystic ameloblastomatoid cystic craniopharyngioma: pathological discussion and clinical significance of cyst formation in adamantinomatous craniopharyngioma. Pediatr Neurosurg (2016) 51(3):158–63. doi: 10.1159/000442992 26795029

[27] Burger P Scheithauer B Vogel F. Surgical pathology of the nervous system and its coverings. 4th edn. Philadelphia: Churchill Livingstone (2002).

[28] Schmalisch K Beschorner R Psaras T Honegger J. Postoperative intracranial seeding of craniopharyngiomas–report of three cases and review of the literature. Acta neurochirurgica (2010) 152(2):313–9. doi: 10.1007/s00701-009-0538-4 19859655

[29] Yang Y Shrestha D Shi XE Zhou Z Qi X Qian H. Ectopic recurrence of craniopharyngioma: Reporting three new cases. Br J Neurosurg (2015) 29(2):295–7. doi: 10.3109/02688697.2014.967751 25311042

[30] Gabel BC Cleary DR Martin JR Khan U Snyder V Sang U H. Unusual and rare locations for craniopharyngiomas: clinical significance and review of the literature. World Neurosurg (2017) 98:381–7. doi: 10.1016/j.wneu.2016.10.134 27908738

[31] Renfrow JJ Greeneway GP Carter L Couture DE. Intraventricular recurrence of a craniopharyngioma: case report. J neurosurgery. Pediatr (2018) 22(4):393–6. doi: 10.3171/2018.4.PEDS18112 29957141

[32] Rachinger W Oehlschlaegel F Kunz M Fuetsch M Schichor C Thurau S. Cystic craniopharyngiomas: microsurgical or stereotactic treatment? Neurosurgery (2017) 80(5):733–43. doi: 10.1227/NEU.0000000000001408 27973392

[33] Piloni M Gagliardi F Bailo M Losa M Boari N Spina A. Craniopharyngioma in pediatrics and adults. Adv Exp Med Biol (2023) 1405:299–329. doi: 10.1007/978-3-031-23705-8_11 37452943

[34] Prieto R Pascual JM Barrios L. Optic chiasm distortions caused by craniopharyngiomas: clinical and magnetic resonance imaging correlation and influence on visual outcome. World Neurosurg (2015) 83(4):500–29. doi: 10.1016/j.wneu.2014.10.002 25308925

[35] Hollon TC Savastano LE Altshuler D Barkan AL Sullivan SE. Ventriculoscopic surgery for cystic retrochiasmatic craniopharyngiomas: indications, surgical technique, and short-term patient outcomes. Operative Neurosurg (Hagerstown Md.) (2018) 15(2):109–19. doi: 10.1093/ons/opx220 29048572

[36] Takano S Akutsu H Mizumoto M Yamamoto T Tsuboi K Matsumura A. Neuroendoscopy followed by radiotherapy in cystic craniopharyngiomas–a long-term follow-up. World Neurosurg (2015) 84(5):1305–15.e152. doi: 10.1016/j.wneu.2015.06.022 26100163

[37] Lauretti L Legninda Sop FY Pallini R Fernandez E D'Alessandris QG. Neuroendoscopic treatment of cystic craniopharyngiomas: A case series with systematic review of the literature. World Neurosurg (2018) 110:e367–73. doi: 10.1016/j.wneu.2017.11.004 29133004

[38] Mohd-Ilham IM Ahmad-Kamal GR Wan Hitam WH Shatriah I. Visual presentation and factors affecting visual outcome in children with craniopharyngioma in east coast states of peninsular Malaysia: A five-year review. Cureus (2019) 11(4):e4407. doi: 10.7759/cureus.4407 31205829 PMC6561515

[39] Wan MJ Zapotocky M Bouffet E Bartels U Kulkarni AV Drake JM. Long-term visual outcomes of craniopharyngioma in children. J neuro-oncology (2018) 137(3):645–51. doi: 10.1007/s11060-018-2762-3 29344823

[40] Chen C Okera S Davies PE Selva D Crompton JL. Craniopharyngioma: a review of long-term visual outcome. Clin Exp Ophthalmol (2003) 31(3):220–8. doi: 10.1046/j.1442-9071.2003.00648.x 12786772

[41] Qiao N Yang X Li C Ma G Kang J Liu C. The predictive value of intraoperative visual evoked potential for visual outcome after extended endoscopic endonasal surgery for adult craniopharyngioma. J Neurosurg (2021) 135(6):1714–24. doi: 10.3171/2020.10.JNS202779 33962373

[42] Zhou Z Zhang S Hu F. Endocrine disorder in patients with craniopharyngioma. Front Neurol (2021) 12:737743. doi: 10.3389/fneur.2021.737743 34925209 PMC8675636

[43] Van Effenterre R Boch AL. Craniopharyngioma in adults and children: a study of 122 surgical cases. J Neurosurg (2002) 97(1):3–11. doi: 10.3171/jns.2002.97.1.0003 12134929

[44] Elliott RE Jane JAJR Wisoff JH. Surgical management of craniopharyngiomas in children: meta-analysis and comparison of transcranial and transsphenoidal approaches. Neurosurgery (2011) 69(3):630–43. doi: 10.1227/NEU.0b013e31821a872d 21499159

[45] Muller HL. Childhood craniopharyngioma. Recent advances in diagnosis, treatment and follow-up. Hormone Res (2008) 69(4):193–202. doi: 10.1159/000113019 18204266

[46] Hoffman HJ De Silva M Humphreys RP Drake JM Smith ML Blaser SI. Aggressive surgical management of craniopharyngiomas in children. J Neurosurg (1992) 76(1):47–52. doi: 10.3171/jns.1992.76.1.0047 1727168

[47] Mende KC Kellner T Petersenn S Honegger J Evangelista-Zamora R Droste M. Clinical situation, therapy, and follow-up of adult craniopharyngioma. J Clin Endocrinol Metab (2020) 105(1):dgz043. doi: 10.1210/clinem/dgz043 31589293

[48] Park YS Chang JH Park YG Kim DS. Recurrence rates after neuroendoscopic fenestration and Gamma Knife surgery in comparison with subtotal resection and Gamma Knife surgery for the treatment of cystic craniopharyngiomas. J Neurosurg (2011) 114(5):1360–8. doi: 10.3171/2009.9.JNS09301 19877807

[49] Eveslage M Calaminus G Warmuth-Metz M Kortmann RD Pohl F Timmermann B. The postopera tive quality of life in children and adolescents with craniopharyngioma. Deutsches Arzteblatt Int (2019) 116(18):321–8. doi: 10.3238/arztebl.2019.0321 PMC6620763 31219033

[50] Hetelekidis S Barnes PD Tao ML Fischer EG Schneider L Scott RM. 20-year experience in childhood craniopharyngioma. Int J Radiat oncology biology Phys (1993) 27(2):189–95. doi: 10.1016/0360-3016(93)90227-m 8407391

[51] Sun F Sun X Du X Xing H Yang B. Factors related to endocrine changes and hormone substitution treatment during pre- and post-operation stages in craniopharyngioma. Oncol Lett (2017) 13(1):250–2. doi: 10.3892/ol.2016.5418 PMC5244892 28123549

[52] Chakrabarti I Amar AP Couldwell W Weiss MH. Long-term neurological, visual, and endocrine outcomes following transnasal resection of craniopharyngioma. J Neurosurg (2005) 102(4):650–7. doi: 10.3171/jns.2005.102.4.0650 15871507

[53] Marx S Tsavdaridou I Paul S Steveling A Schirmer C Eördögh M. Quality of life and olfactory function after suprasellar craniopharyngioma surgery-a single-center experience comparing transcranial and endoscopic endonasal approaches. Neurosurgical Rev (2021) 44(3):1569–82. doi: 10.1007/s10143-020-01343-x PMC8121742 32651708

[54] Schreckinger M Walker B Knepper J Hornyak M Hong D Kim JM. Post-operative diabetes insipidus after endoscopic transsphenoidal surgery. Pituitary (2013) 16(4):445–51. doi: 10.1007/s11102-012-0453-1 23242859

[55] Chen A Zhou R Yao X Tong Z Li J Xiang R. Neuroendoscopic surgery combined with Ommaya reservoir placement for cystic craniopharyngiomas: 11 years of experience in a single institution. Br J Neurosurg (2022), 1–7. doi: 10.1080/02688697.2022.2152776 36469601

[56] Young SC Zimmerman RA Nowell MA Bilaniuk LT Hackney DB Grossman RI. Giant cystic craniopharyngiomas. Neuroradiology (1987) 29(5):468–73. doi: 10.1007/BF00341745 3317110

[57] Chen A Zhou R Yao X Ai M Sun T. Neuroendoscopic treatment of giant cystic craniopharyngioma in the foramen magnum: report of two cases. Child's nervous system (2021) 37(7):2387–90. doi: 10.1007/s00381-020-04965-0 33169209

[58] Kiran NA Suri A Kasliwal MK Garg A Ahmad FU Mahapatra AK. Gross total excision of pediatric giant cystic craniopharyngioma with huge retroclival extension to the level of foramen magnum by anterior trans petrous approach: report of two cases and review of literature. Child's nervous system (2008) 24(3):385–91. doi: 10.1007/s00381-007-0522-3 18034348

[59] Connolly J.R. ES Winfree CJ Carmel PW. Giant posterior fossa cystic craniopharyngiomas presenting with hearing loss. Report of three cases and review of the literature. Surg Neurol (1997) 47(3):291–9. doi: 10.1016/s0090-3019(96)00253-4 9068702

[60] Zhao R Deng J Liang X Zeng J Chen X Wang J. Treatment of cystic craniopharyngioma with phosphorus-32 intracavitary irradiation. Child's nervous system (2010) 26(5):669–74. doi: 10.1007/s00381-009-1025-1 PMC3617497 19904543

[61] Moorthy RK Backianathan S Rebekah G Rajshekhar V. Utility of interval imaging during focused radiation therapy for residual cystic craniopharyngiomas. World Neurosurg (2020) 141:e615–24. doi: 10.1016/j.wneu.2020.05.258 32522649

[62] Buhl R Lang EW Barth H Mehdorn HM. Giant cystic craniopharyngiomas with extension into the posterior fossa. Child's nervous system (2000) 16(3):138–42. doi: 10.1007/s003810050480 10804048

[63] Goyal A Singh AK Sinha S. Giant cystic craniopharyngioma with posterior fossa extension. Pediatr Neurosurg (2002) 37(1):50–1. doi: 10.1159/000065103 12138221

[64] Gangemi M Seneca V Mariniello G Colella G Magro F. Combined endoscopic and microsurgical removal of a giant cystic craniopharyngioma in a six-year-old boy. Clin Neurol Neurosurg (2009) 111(5):472–6. doi: 10.1016/j.clineuro.2009.01.002 19200643

[65] Sener RN Kismali E Akyar S Selcuki M Yalman O. Large craniopharyngioma extending to the posterior cranial fossa. Magnetic resonance Imaging (1997) 15(9):1111–2. doi: 10.1016/s0730-725x(97)00137-9 9364961

[66] Akinduro OO Izzo A Lu VM Ricciardi L Trifiletti D Peterson JL. Endocrine and visual outcomes following gross total resection and subtotal resection of adult craniopharyngioma: systematic review and meta-analysis. World Neurosurg (2019) 127:e656–68. doi: 10.1016/j.wneu.2019.03.239 30947004

[67] Locatelli D Massimi L Rigante M Custodi V Paludetti G Castelnuovo P. Endoscopic endonasal transsphenoidal surgery for sellar tumors in children. Int J Pediatr otorhinolaryngology (2010) 74(11):1298–302. doi: 10.1016/j.ijporl.2010.08.009 20828839

[68] Ceylan S Caklili M Emengen A Yilmaz E Anik Y Selek A. An endoscopic endonasal approach to craniopharyngioma via the infrachiasmatic corridor: a single center experience of 84 patients. Acta neurochirurgica (2021) 163(8):2253–68. doi: 10.1007/s00701-021-04832-0 33830341

[69] Fong RP Babu CS Schwartz TH. Endoscopic endonasal approach for craniopharyngiomas. J neurosurgical Sci (2021) 65(2):133–9. doi: 10.23736/S0390-5616.21.05097-9 33890754

[70] Cossu G Jouanneau E Cavallo LM Elbabaa SK Giammattei L Starnoni D. Surgical management of craniopharyngiomas in adult patients: a systematic review and consensus statement on behalf of the EANS skull base section. Acta neurochirurgica (2020) 162(5):1159–77. doi: 10.1007/s00701-020-04265-1 32112169

[71] Na MK Jang B Choi KS Lim TH Kim W Cho Y. Craniopharyngioma resection by endoscopic endonasal approach versus transcranial approach: A systematic review and meta-analysis of comparative studies. Front Oncol (2022) 12:1058329. doi: 10.3389/fonc.2022.1058329 36530998 PMC9748146

[72] Qiao N Li C Liu F Ru S Cai K Jia Y. Risk factors for cerebrospinal fluid leak after extended endoscopic endonasal surgery for adult patients with craniopharyngiomas: a multivariate analysis of 364 cases. J Neurosurg (2023), 1–12. doi: 10.3171/2023.5.JNS222791 37382333

[73] Cavallo LM Frank G Cappabianca P Solari D Mazzatenta D Villa A. The endoscopic endonasal approach for the management of craniopharyngiomas: a series of 103 patients. J Neurosurg (2014) 121(1):100–13. doi: 10.3171/2014.3.JNS131521 24785324

[74] Zhou Y Wei J Jin T Hei Y Jia P Lin J. Extended endoscopic endonasal approach for resecting anterior intrinsic third ventricular craniopharyngioma. Front Oncol (2022) 12:998683. doi: 10.3389/fonc.2022.998683 36248957 PMC9562125

[75] Shoubash LI El Refaee E Al Menabbawy A Refaat MI Fathalla H Schroeder HWS. Endoscopic transcortical-transventricular approach in treating third ventricular craniopharyngiomas-case series with technical note and literature review. Operative Neurosurg (Hagerstown Md.) (2022) 22(4):192–200. doi: 10.1227/ONS.0000000000000114 35147594

[76] Cavallo LM Solari D Esposito F Cappabianca P. The endoscopic endonasal approach for the management of craniopharyngiomas involving the third ventricle. Neurosurgical Rev (2013) 36(1):27–38. doi: 10.1007/s10143-012-0403-4 22791074

[77] Steiert C Grauvogel J Roelz R Demerath T Schnell D Beck J. Stereotactic cysto-ventricular catheters in craniopharyngiomas: an effective minimally invasive method to improve visual impairment and achieve long-term cyst volume reduction. Neurosurgical Rev (2021) 44(6):3411–20. doi: 10.1007/s10143-021-01510-8 PMC8592958 33674982

[78] Delitala A Brunori A Chiappetta F. Purely neuroendoscopic transventricular management of cystic craniopharyngiomas. Child's nervous system (2004) 20(11-12):858–62. doi: 10.1007/s00381-004-0943-1 15322844

[79] Tirakotai W Schulte DM Bauer BL Bertalanffy H Hellwig D. Neuroendoscopic surgery of intracranial cysts in adults. Child's nervous system (2004) 20(11-12):842–51. doi: 10.1007/s00381-004-0941-3 15197568

[80] Schubert T Trippel M Tacke U van Velthoven V Gumpp V Bartelt S. Neurosurgical treatment strategies in childhood craniopharyngiomas: is less more? Child's nervous system (2009) 25(11):1419–27. doi: 10.1007/s00381-009-0978-4 19714341

[81] Moussa AH Kerasha AA Mahmoud ME. Surprising outcome of ommaya reservoir in treating cystic craniopharyngioma: a retrospective study. Br J Neurosurg (2013) 27(3):370–3. doi: 10.3109/02688697.2012.741732 23167666

[82] Frio F Solari D Cavallo LM Cappabianca P Raverot G Jouanneau E. Ommaya reservoir system for the treatment of cystic craniopharyngiomas: surgical results in a series of 11 adult patients and review of the literature. World Neurosurg (2019) 132:e869–77. doi: 10.1016/j.wneu.2019.07.217 31400528

[83] Wang A Tenner MS Tobias ME Mohan A Kim D Tandon A. A novel approach using electromagnetic neuronavigation and a flexible neuroendoscope for placement of ommaya reservoirs. World Neurosurg (2016) 96:195–201. doi: 10.1016/j.wneu.2016.08.127 27609447

[84] Mori R Joki T Nonaka Y Ikeuchi S Abe T. Parallel insertion endoscopic technique for precise catheter placement in cystic craniopharyngiomas. J neurological surgery. Part A Cent Eur Neurosurg (2014) 75(6):442–6. doi: 10.1055/s-0033-1349341 23959614

[85] Vitaz TW Hushek S Shields CB Moriarty T. Changes in cyst volume following intraoperative MRI-guided Ommaya reservoir placement for cystic craniopharyngioma. Pediatr Neurosurg (2001) 35(5):230–4. doi: 10.1159/000050427 11741115

[86] Liu X Yu Q Zhang Z Zhang Y Li Y Liu D. Same-day stereotactic aspiration and Gamma Knife surgery for cystic intracranial tumors. J Neurosurg (2012) 117 Suppl:45–8. doi: 10.3171/2012.7.GKS121019 23205788

[87] Lau JC Kosteniuk SE Walker T Iansavichene A Macdonald DR Megyesi JF. Operative complications with and without image guidance: A systematic review and meta-analysis of the ommaya reservoir literature. World Neurosurg (2019) 122:404–14. doi: 10.1016/j.wneu.2018.11.036 30447448

[88] Pettorini BL Tamburrini G Massimi L Caldarelli M Di Rocco C. Endoscopic transventricular positioning of intracystic catheter for treatment of craniopharyngioma. Tech note. J neurosurgery. Pediatr (2009) 4(3):245–8. doi: 10.3171/2009.4.PEDS0978 19772408

[89] Sampath R Wadhwa R Tawfik T Nanda A Guthikonda B. Stereotactic placement of ventricular catheters: does it affect proximal malfunction rates? Stereotactic Funct Neurosurg (2012) 90(2):97–103. doi: 10.1159/000333831 22398576

[90] Wang G Zhang X Feng M Guo F. Comparing survival outcomes of gross total resection and subtotal resection with radiotherapy for craniopharyngioma: a meta-analysis. J Surg Res (2018) 226:131–9. doi: 10.1016/j.jss.2018.01.029 29661278

[91] Schoenfeld A Pekmezci M Barnes MJ Tihan T Gupta N Lamborn KR. The superiority of conservative resection and adjuvant radiation for craniopharyngiomas. J neuro-oncology (2012) 108(1):133–9. doi: 10.1007/s11060-012-0806-7 PMC3879154 22350375

[92] Dandurand C Sepehry AA Asadi Lari MH Akagami R Gooderham P. Adult craniopharyngioma: case series, systematic review, and meta-analysis. Neurosurgery (2018) 83(4):631–41. doi: 10.1093/neuros/nyx570 29267973

[93] Clark AJ Cage TA Aranda D Parsa AT Sun PP Auguste KI. A systematic review of the results of surgery and radiotherapy on tumor control for pediatric craniopharyngioma. Child's nervous system (2013) 29(2):231–8. doi: 10.1007/s00381-012-1926-2 23089933

[94] Yang I Sughrue ME Rutkowski MJ Kaur R Ivan ME Aranda D. Craniopharyngioma: a comparison of tumor control with various treatment strategies. Neurosurgical Focus (2010) 28(4):E5. doi: 10.3171/2010.1.FOCUS09307 20367362

[95] Merchant TE Hoehn ME Khan RB Sabin ND Klimo P Boop FA. Proton therapy and limited surgery for paediatric and adolescent patients with craniopharyngioma (RT2CR): a single-arm, phase 2 study. Lancet Oncol (2023) 24(5):523–34. doi: 10.1016/S1470-2045(23)00146-8 PMC10408380 37084748

[96] Bishop AJ Greenfield B Mahajan A Paulino AC Okcu MF Allen PK. Proton beam therapy versus conformal photon radiation therapy for childhood craniopharyngioma: multi-institutional analysis of outcomes, cyst dynamics, and toxicity. Int J Radiat oncology biology Phys (2014) 90(2):354–61. doi: 10.1016/j.ijrobp.2014.05.051 PMC4194252 25052561

[97] Winkfield KM Linsenmeier C Yock TI Grant PE Yeap BY Butler WE. Surveillance of craniopharyngioma cyst growth in children treated with proton radiotherapy. Int J Radiat oncology biology Phys (2009) 73(3):716–21. doi: 10.1016/j.ijrobp.2008.05.010 18676089

[98] Lee CC Yang HC Chen CJ Hung YC Wu HM Shiau CY. Gamma Knife surgery for craniopharyngioma: report on a 20-year experience. J Neurosurg (2014) 121 Suppl:167–78. doi: 10.3171/2014.8.GKS141411 25434950

[99] Chung WY Pan DH Shiau CY Guo WY Wang LW. Gamma knife radiosurgery for craniopharyngiomas. J Neurosurg (2000) 93 Suppl 3:47–56. doi: 10.3171/jns.2000.93.supplement 11143262

[100] Julow J Backlund EO Lányi F Hajda M Bálint K Nyáry I. Long-term results and late complications after intracavitary yttrium-90 colloid irradiation of recurrent cystic craniopharyngiomas. Neurosurgery (2007) 61(2):288–96. doi: 10.1227/01.NEU.0000255528.68963.EF 17762741

[101] Prasad D Steiner M Steiner L. Gamma knife surgery for craniopharyngioma. Acta neurochirurgica (1995) 134(3-4):167–76. doi: 10.1007/BF01417685 8748777

[102] Guimarães MM Cardeal DD Teixeira MJ Lucio JEDC Sanders FH Kuromoto RK. Brachytherapy in paediatric craniopharyngiomas: a systematic review and meta-analysis of recent literature. Child's nervous system (2022) 38(2):253–62. doi: 10.1007/s00381-021-05378-3 34618201

[103] Yu X Christ SM Liu R Wang Y Hu C Feng B. Evaluation of long-term outcomes and toxicity after stereotactic phosphorus-32-based intracavitary brachytherapy in patients with cystic craniopharyngioma. Int J Radiat oncology biology Phys (2021) 111(3):773–84. doi: 10.1016/j.ijrobp.2021.05.123 34058257

[104] Hasegawa T Kondziolka D Hadjipanayis CG Lunsford LD. Management of cystic craniopharyngiomas with phosphorus-32 intracavitary irradiation. Neurosurgery (2004) 54(4):813–22. doi: 10.1227/01.neu.0000114262.30035.af 15046646

[105] Kickingereder P Maarouf M El Majdoub F Fuetsch M Lehrke R Wirths J. Intracavitary brachytherapy using stereotactically applied phosphorus-32 colloid for treatment of cystic craniopharyngiomas in 53 patients. J neuro-oncology (2012) 109(2):365–74. doi: 10.1007/s11060-012-0902-8 22717668

[106] Pollack IF Lunsford LD Slamovits TL Gumerman LW Levine G Robinson AG. Stereotaxic intracavitary irradiation for cystic craniopharyngiomas. J Neurosurg (1988) 68(2):227–33. doi: 10.3171/jns.1988.68.2.0227 3276836

[107] Pollock BE Lunsford LD Kondziolka D Levine G Flickinger JC. Phosphorus-32 intracavitary irradiation of cystic craniopharyngiomas: current technique and long-term results. Int J Radiat oncology biology Phys (1995) 33(2):437–46. doi: 10.1016/0360-3016(95)00175-X 7673031

[108] Shahzadi S Sharifi G Andalibi R Zali A Ali-Asgari A. Management of cystic craniopharyngiomas with intracavitary irradiation with 32P. Arch Iranian Med (2008) 11(1):30–4. 18154420

[109] Barriger RB Chang A Lo SS Timmerman RD DesRosiers C Boaz JC. Phosphorus-32 therapy for cystic craniopharyngiomas. Radiotherapy Oncol (2011) 98(2):207–12. doi: 10.1016/j.radonc.2010.12.001 21269713

[110] Yu X Zhang J Liu R Wang Y Wang H Wang P. Interstitial radiotherapy using phosphorus-32 for giant posterior fossa cystic craniopharyngiomas. J neurosurgery. Pediatr (2015) 15(5):510–8. doi: 10.3171/2014.10.PEDS14302 25679384

[111] Maarouf M El Majdoub F Fuetsch M Hoevels M Lehrke R Berthold F. Stereotactic intracavitary brachytherapy with P-32 for cystic craniopharyngiomas in children. Strahlentherapie und Onkologie (2016) 192(3):157–65. doi: 10.1007/s00066-015-0910-7 26541336

[112] Mottolese C Stan H Hermier M Berlier P Convert J Frappaz D. Intracystic chemotherapy with bleomycin in the treatment of craniopharyngiomas. Child's nervous system (2001) 17(12):724–30. doi: 10.1007/s00381-001-0524-5 11862438

[113] Hukin J Steinbok P Lafay-Cousin L Hendson G Strother D Mercier C. Intracystic bleomycin therapy for craniopharyngioma in children: the Canadian experience. Cancer (2007) 109(10):2124–31. doi: 10.1002/cncr.22633 17407137

[114] Mottolese C Szathmari A Berlier P Hermier M. Craniopharyngiomas: our experience in Lyon. Child's nervous system (2005) 21(8-9):790–8. doi: 10.1007/s00381-005-1242-1 15971075

[115] Lafay-Cousin L Bartels U Raybaud C Kulkarni AV Guger S Huang A. Neuroradiological findings of bleomycin leakage in cystic craniopharyngioma. Rep three cases. J Neurosurg (2007) 107(4 Suppl):318–23. doi: 10.3171/PED-07/10/318 17941498

[116] Haisa T Ueki K Yoshida S. Toxic effects of bleomycin on the hypothalamus following its administration into a cystic craniopharyngioma. Br J Neurosurg (1994) 8(6):747–50. doi: 10.3109/02688699409101192 7536420

[117] Savas A Erdem A Tun K Kanpolat Y. Fatal toxic effect of bleomycin on brain tissue after intracystic chemotherapy for a craniopharyngioma: case report. Neurosurgery (2000) 46(1):213–7. doi: 10.1097/00006123-200001000-00043 10626953

[118] Liu W Fang Y Cai B Xu J You C Zhang H. Intracystic bleomycin for cystic craniopharyngiomas in children (abridged republication of cochrane systematic review). Neurosurgery (2012) 71(5):909–15. doi: 10.1227/NEU.0b013e31826d5c31 22902333

[119] Zhang S Fang Y Cai BW Xu JG You C. Intracystic bleomycin for cystic craniopharyngiomas in children. Cochrane Database systematic Rev (2016) 7(7):CD008890. doi: 10.1002/14651858.CD008890.pub4 PMC6457977 27416004

[120] Zheng J Fang Y Cai BW Zhang H Liu W Wu B. Intracystic bleomycin for cystic craniopharyngiomas in children. Cochrane Database systematic Rev (2014) 9):CD008890. doi: 10.1002/14651858.CD008890.pub3 25233847

[121] Mrowczynski OD Langan ST Rizk EB. Craniopharyngiomas: A systematic review and evaluation of the current intratumoral treatment landscape. Clin Neurol Neurosurg (2018) 166:124–30. doi: 10.1016/j.clineuro.2018.01.039 29408768

[122] Cavalheiro S Di Rocco C Valenzuela S Dastoli PA Tamburrini G Massimi L. Craniopharyngiomas: intratumoral chemotherapy with interferon-alpha: a multicenter preliminary study with 60 cases. Neurosurgical Focus (2010) 28(4):E12. doi: 10.3171/2010.1.FOCUS09310 20367356

[123] Kilday JP Caldarelli M Massimi L Chen RH Lee YY Liang ML. Intracystic interferon-alpha in pediatric craniopharyngioma patients: an international multicenter assessment on behalf of SIOPE and ISPN. Neuro-oncology (2017) 19(10):1398–407. doi: 10.1093/neuonc/nox056 PMC5596165 28499018

[124] Gan HW Morillon P Albanese A Aquilina K Chandler C Chang YC. National UK guidelines for the management of paediatric craniopharyngioma. Lancet Diabetes Endocrinol (2023) 11(9):694–706. doi: 10.1016/S2213-8587(23)00162-6 37549682

[125] Hölsken A Gebhardt M Buchfelder M Fahlbusch R Blümcke I Buslei R. EGFR signaling regulates tumor cell migration in craniopharyngiomas. Clin Cancer research: an Off J Am Assoc Cancer Res (2011) 17(13):4367–77. doi: 10.1158/1078-0432.CCR-10-2811 21562037

[126] Patel K Allen J Zagzag D Wisoff J Radmanesh A Gindin T. Radiologic response to MEK inhibition in a patient with a WNT-activated craniopharyngioma. Pediatr Blood Cancer (2021) 68(3):e28753. doi: 10.1002/pbc.28753 33073916

[127] Grob S Mirsky DM Donson AM Dahl N Foreman NK Hoffman LM. Targeting IL-6 is a potential treatment for primary cystic craniopharyngioma. Front Oncol (2019) 9:791. doi: 10.3389/fonc.2019.00791 31497533 PMC6712354

[128] Mollá E Martí-Bonmatí L Revert A Arana E Menor F Dosdá R. Craniopharyngiomas: identification of different semiological patterns with MRI. Eur Radiol (2002) 12(7):1829–36. doi: 10.1007/s00330-001-1196-y 12111075

[129] d'Avella E Vitulli F Berardinelli J Cinalli G Solari D Cappabianca P. Systematic review of transcranial and endoscopic endonasal approaches for craniopharyngiomas in children: is there an evolution? J neurosurgery. Pediatr (2023), 1–12. doi: 10.3171/2023.9.PEDS23117 37948683

[130] Mazzatenta D Zoli M Guaraldi F Ambrosi F Faustini Fustini M Pasquini E. Outcome of endoscopic endonasal surgery in pediatric craniopharyngiomas. World Neurosurg (2020) 134:e277–88. doi: 10.1016/j.wneu.2019.10.039 31629927

[131] Wu J Pan C Xie S Tang B Fu J Wu X. A propensity-adjusted comparison of endoscopic endonasal surgery versus transcranial microsurgery for pediatric craniopharyngioma: a single-center study. J neurosurgery. Pediatr (2021) 29(3):325–34. doi: 10.3171/2021.10.PEDS21392 34920437

[132] Sterkenburg AS Hoffmann A Gebhardt U Warmuth-Metz M Daubenbüchel AM Müller HL. Survival, hypothalamic obesity, and neuropsychological/psychosocial status after childhood-onset craniopharyngioma: newly reported long-term outcomes. Neuro-oncology (2015) 17(7):1029–38. doi: 10.1093/neuonc/nov044 PMC5654354 25838139

[133] Bogusz A Müller HL. Childhood-onset craniopharyngioma: latest insights into pathology, diagnostics, treatment, and follow-up. Expert Rev Neurother (2018) 18(10):793–806. doi: 10.1080/14737175.2018.1528874 30257123

[134] Ma G Kang J Qiao N Zhang B Chen X Li G. Non-invasive radiomics approach predict invasiveness of adamantinomatous craniopharyngioma before surgery. Front Oncol (2021) 10:599888. doi: 10.3389/fonc.2020.599888 33680925 PMC7925821

